# Trade-offs among transport, support, and storage in xylem from shrubs in a semiarid chaparral environment tested with structural equation modeling

**DOI:** 10.1073/pnas.2104336118

**Published:** 2021-08-13

**Authors:** R. B. Pratt, A. L. Jacobsen, M. I. Percolla, M. E. De Guzman, C. A. Traugh, M. F. Tobin

**Affiliations:** ^a^Department of Biology, California State University, Bakersfield, CA 93311;; ^b^Department of Biology, University of Houston, Downtown, Houston, TX 77002

**Keywords:** biomechanics, capacitance, carbohydrates, cavitation, drought

## Abstract

The xylem in plants is specialized to transport water, mechanically support the plant body, and store water and carbohydrates. Balancing these functions leads to trade-offs that are linked to xylem structure. We proposed a multivariate hypothesis regarding the main xylem functions and tested it using structural equation modeling. We sampled 29 native shrub species from field sites in semiarid Southern California. We quantified xylem water transport (embolism resistance and transport efficiency), mechanical strength, storage of water (capacitance) and starch, minimum hydrostatic pressures (*P*_min_), and proportions of fibers, vessels, and parenchyma, which were treated as a latent variable representing “cellular trade-offs.” We found that xylem functions (transport, mechanical support, water storage, and starch storage) were independent, a result driven by *P*_min_. *P*_min_ was strongly and directly or indirectly associated with all xylem functions as a hub trait. More negative *P*_min_ was associated with increased embolism resistance and tissue strength and reduced capacitance and starch storage. We found strong support for a trade-off between embolism resistance and transport efficiency. Tissue strength was not directly associated with embolism resistance or transport efficiency, and any associations were indirect involving *P*_min_. With *P*_min_ removed from the model, cellular trade-offs were central and related to all other traits. We conclude that xylem traits are broadly governed by functional trade-offs and that the *P*_min_ experienced by plants in the field exerts a strong influence over these relationships. Angiosperm xylem contains different cell types that contribute to different functions and that underpin trade-offs.

## Significance

Plant vascular systems play a central role in global water and carbon cycles and drought resistance. These vascular systems perform multiple functions that affect the fitness of plants, and trade-offs are present among these functions. Some trade-offs are well established, but studies have not examined the full suite of functions of these complex systems. Here, we used a powerful multivariate method, structural equation modeling, to test hypotheses about the trade-offs that govern this vital and globally important tissue. We show that xylem traits are broadly governed by trade-offs related to transport, mechanical support, and storage, which are rooted in cellular structure, and that the level of dehydration experienced by plants in the field exerts a strong influence over these relationships.

From cells to ecosystems, biological systems are complex and span multiple scales. To fully understand such systems, multivariate analytical methods are a powerful tool (1), yet it is most common to analyze variables separately or descriptively ordinate them. One powerful multivariate analytical framework is structural equation modeling (SEM) (2, 3). Plant vascular systems represent a complex multivariate system, where many traits determine functions in direct and indirect ways and interact with one another. There is much interest in understanding xylem in a systems context (4–6); however, using SEM to test hypotheses of the full range of xylem function in a single model has yet to be done. A positive development is that several recent studies that have applied SEM to understanding some xylem functions and traits (7–9).

Xylem functions include transport of water, mechanical support, and storage of water and carbohydrates (reviewed in ref. 6). These functions are interrelated, and associations among traits arise due to mechanistic links between structure and function. This can lead to trade-offs where prowess in one trait necessarily diminishes that of another (10). Traits may also be associated for at least two other reasons: shared ancestry, or when ecological conditions select for a suite of adaptive traits in different lineages in a process called concerted convergence (11).

**Fig. 1.**
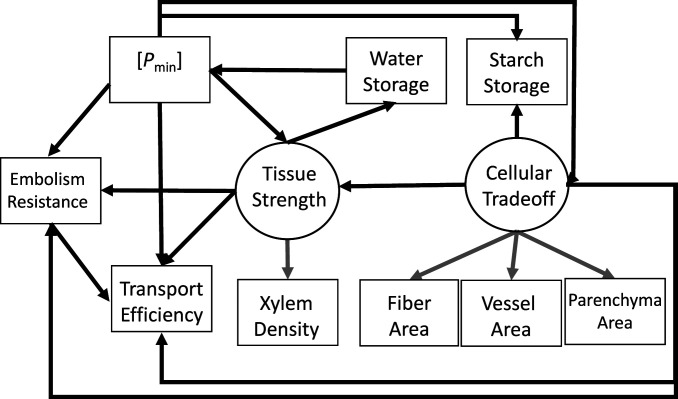
Hypothesized relationships among the various xylem functions. The arrows represent pathways between two variables. Traits may have direct effects on another trait represented by an arrow directly connecting two traits, and traits may also exert indirect effects when they are connected through an intermediate trait. Latent variables are connected to their measured traits by gray arrows. Cellular trade-off is a latent variable represented by measured fiber, parenchyma, and vessel area in cross section. Tissue strength is represented by xylem density. Omitted are any double-headed arrows for variables with correlated errors (*SI Appendix*).

We present relationships among xylem traits as a multivariate hypothesis in a path diagram (Fig. 1). The path model depicts the multiple variables, and the arrows (paths) represent connections between variables that can be direct (a direct arrow from one to another) or indirect (a direct arrow to a trait that has a direct path to a second trait), where indirect effects can be as important as direct ones. There are two central elements to our hypothesized model. First, that there are different cell types that are specialized to perform xylem functions: vessels conduct water; fibers provide support; and parenchyma stores carbohydrates (see also ref. 12). The division of cellular labor mitigates some direct functional trade-offs found in species with tracheid-based vascular systems (13); nevertheless, trade-offs may arise based on the amount of tissue volume allocated to different cells (4, 14). We examined this trade-off as a latent variable in our SEM model where “cellular trade-off” is represented by the proportions of different cell types in cross section (Fig. 1). The second centerpiece in our hypothesis is that the hydrostatic pressure potential experienced by plants during droughts or dry periods (*P*_min_) exerts a mechanical strain giving rise to direct and indirect effects on all other traits (11, 15, 16). This trait is affected by the environment (amount and timing of rainfall, temperature, and soil water content and conductance), and plant traits such as water use and hydraulic conductance, with additional links to many other traits (11).

## Rationale for Our Hypothesized Relationships

We hypothesized that *P*_min_ is directly associated with embolism resistance and indirectly affects hydraulic transport efficiency (Fig. 1). Emboli are gas bubbles that form in conduits that block transport during drought or following freeze–thaw events (4). Species have evolved broad differences in embolism resistance (xylem safety) that is under strong selection by drought when negative pressures in the xylem exceed safety thresholds, leading to dieback and mortality (17–19); moreover, xylem safety is strongly associated with *P*_min_ (20, 21).

Increased embolism resistance is directly linked to reduced hydraulic transport efficiency in a well-studied trade-off (9, 22). Efficiency of xylem refers to the mass flow rate of water for a given pressure gradient and area of tissue (xylem-specific conductivity, *K*_s_). No species has xylem that is simultaneously highly resistant to embolism (very safe) and highly efficient (23). One reason for this is because of the pits and pit membranes that connect conduits. These cellulosic membranes have nanoscopic pores, and the smaller these pores, the more resistant they are to embolism spread; however, smaller pores and thicker membranes reduce transport efficiency (22, 24). The arrangement and connections of the network of vessels in a vascular system is also an important factor (25). Globally and across angiosperm and gymnosperm lineages, a safety–efficiency trade-off has not been supported (16); however, within a specific lineage, community, or growth form, this trade-off can occur, and understanding this context is a research priority (9, 10). *P*_min_ and efficiency are additionally predicted to be directly related because of an effect of *P*_min_ on vessel diameters. Larger-diameter vessels are associated with greater efficiency (26), and such vessels can take longer to develop (27). If water is limited when vessels are developing, then the diminished turgor will limit vessel size (28).

Cellular trade-offs and tissue strength may be directly affected by *P*_min_ because extreme pressures can strain conduits to the point of buckling damage or collapse (29). This threat is minimized by thicker cell walls between conduits, smaller conduit diameters, and an extensive and supportive fiber matrix (13, 30, 31), all of which create a series of direct and indirect paths (Fig. 1). First, these factors should lead to direct associations between *P*_min_ and tissue strength (4, 29, 32) and with cellular trade-offs. A cellular trade-off affects tissue strength because more fibers promote strength at the expense of parenchyma and vessels (6). The association between tissue strength and *P*_min_ creates four indirect paths from tissue strength (Fig. 1). These pathways lead to associations with embolism resistance and efficiency and that are hypothesized to arise because the more negative *P*_min_ a plant experiences, the greater the need for vessels to resist embolism (30). Additionally, efficiency is reduced because smaller-diameter vessels better resist implosion (29), and stronger vessel walls are thicker and create deeper and longer pit chambers (24). Another indirect association is between tissue strength and water storage capacity. For nonsucculent woody species, most water is stored in the lumens of fibers and stronger tissues with thick-walled fibers, and narrow lumens have lower water storage (33). A final indirect association is predicted between tissue strength and *P*_min_ through its effect on water storage, which leads to a feedback loop among these three traits.

Storage of carbohydrates in xylem allows plants to cope with variable and uncertain environments (34). Their diverse functional roles are an area of active research (34), and they are important to understand in the context of trade-offs (6, 8). Stored carbohydrates are found in parenchyma, thus increased storage capacity requires an increase in these cells [living fibers can also be important (35, 36)], which links cellular trade-offs to carbohydrate storage. Parenchyma may be structurally diverse, but they are generally thin-walled living cells that provide the least support to vessels in resisting implosion and mechanical strains contributing to the link between cellular trade-off and tissue strength and embolism resistance (30, 37). *P*_min_ is hypothesized to be directly linked to starch storage because species that experience more negative *P*_min_ osmoregulate by hydrolyzing starch to simple sugars (34, 38), which should create a negative association between *P*_min_ and starch storage.

Two other direct trade-offs are predicted between cellular trade-offs and transport efficiency and embolism resistance. Previous work has found an association between the proportion of vessels in xylem (vessel area) and transport efficiency (32) or the proportion of vessel lumen area (39). We also predicted a direct relationship between cellular trade-offs and embolism resistance. This association could arise due to direct associations between proportions of cellular traits and their importance in resisting the strain of negative pressures, or this may simply be indirect through a direct effect on tissue strength. These associations also create the potential for indirect associations of *P*_min_ with transport efficiency and embolism resistance through association with cellular trade-offs.

We used an SEM approach to test our model and hypotheses (represented in Fig. 1). Both cellular trade-offs and *P*_min_ were predicted to affect all other traits directly or indirectly. Evaluating both simultaneously is informative, but to understand how they affected one another, we created an additional model with *P*_min_ removed. Our hypotheses determined the paths in the diagram and the direction of their effects; however, other formulations are possible and are discussed. We measured variables representing different xylem functions and *P*_min_ in 29 species of chaparral shrubs from Southern California. All species were growing at field sites with a semiarid Mediterranean-type climate. This system has a protracted dry season that places considerable strain on vascular transport traits (40); moreover, the values for xylem traits found among chaparral shrubs, even co-occurring ones, span a wide range, providing abundant trait variation (21, 36). All species were sampled in the same laboratory and using the same methods, thus minimizing errors due to methods differences.

## Materials and Methods

Shrub species (*n* = 29) were measured at four field sites in Southern California (*SI Appendix*, Table S1). At all sites, *n* = 6 different individuals were tagged for sampling for each species at that site. Our goal was to study many independent species, thus sites were selected that contained diverse species (mixed chaparral). We also selected those of a similar community type and that contained abundant individuals of the indicator species chamise (*Adenostoma fasciculatum*). In chaparral classification, these sites would be mixed/chamise-type chaparral (40). Sites had not experienced a burn in at least 30 y, so they contained mature shrubs. All sites have a Mediterranean-type climate with hot dry summers and cool moist winters. Precipitation is almost entirely rainfall that occurs between November and May each year, with a protracted rainless season occurring in the Summer and Fall months. For more details on the sites, see ref. 36. Most of the samples and data were collected in 2009 and 2010. The phylogeny of the sampled species was reconstructed using the phylomatic database, and it was fine-tuned using a molecular phylogeny (see ref. 36 for additional details).

### Plant Traits.

We measured a suite of traits to represent xylem functions with an aim to include them in a structural equation model. In many cases, there are multiple traits that could represent a function. Because our goal was to present a simple model, we did not include all the measured traits in our model because it was overly complicated and impractical. In the following sections, we highlight the care that we took to compare methods and measures to ensure the traits we chose represented a particular xylem function. The target sample size for all measurements was *n* = 6 different individuals per species, and the same individuals were used throughout the study to minimize intraspecific error variation. The mean of these six samples was the unit of analysis for species. For all measured traits, we sampled healthy branches that were similarly sized (about 6 mm in diameter) and located in the sunny south side of the outer canopy to minimize branch-to-branch variation. We measured multiple variables on the same stems when possible, which included hydraulic measurements, xylem density, and anatomy. Methods are fully described for most traits. Starch storage and measures of xylem cellular proportions have been previously published (36), and methods for these traits are only briefly described with the relevant publications referenced.

Resistance to embolism of distal branches was measured using a centrifuge method. Samples were brought back to the laboratory and flushed prior to sampling (see next paragraph). This method exposes stems to increasingly negative xylem pressures and measures hydraulic conductivity (*K*_h_) declines in response. The resistance to embolism is expressed as the negative pressure for a given percentage loss of *K*_h_. It is common to use the pressure potential at a loss of 50% of maximum *K*_h_ (*P*_50_). Here, we used the pressure potential at a 75% loss in *K*_h_ (*P*_75_). The *P*_50_ and *P*_75_ were strongly correlated (*r* = 0.91, *P* << 0.001), so this choice did not alter the analyses. The sampling protocol followed methods that have been previously published and extensively compared to reference methods (e.g., ref. 41).

Hydraulic efficiency was measured on the same stems as used for *P*_75_. Stems were 14 cm long and were flushed for 60 min at 100 kPa with an ultrafiltered (0.01-μm pore) and degassed 20 mM KCl solution. The flushing treatment removed emboli from the stem xylem. The stems were then connected to a tubing system with a pressure head of 2 to 3 kPa, and flow through the stem was collected on a four-point balance. The flow rate (kg/ s) was divided by the pressure gradient (MPa/ m) to compute the *K*_h_ of the stems. This was divided by the sapwood area to compute the xylem-specific *K*_h_ (*K*_s_), which is a trait commonly used to represent transport efficiency. Another trait that can represent efficiency is vessel diameter. We compared our *K*_s_ data to vessel diameter to validate them. The *K*_s_ was strongly and positively correlated to vessel diameter (*r* = 0.78, *P* << 0.001).

The minimum seasonal water potential was measured on distal branchlets at the end of the Fall dry season in 2009 using a pressure chamber (Model 2000, PMS Instrument Co.). Not all sites could be sampled before rains fell in 2009, so additional sampling was completed in Fall 2010. Samples were taken at predawn and midday. In theory, the predawn values equilibrate with soil water potential, and all the organs of the plant are in equilibrium including the stem xylem pressure potential (*P*). The predawn and midday values were strongly correlated (*r* = 0.95, *P* << 0.001), and we report midday values as *P*_min_. The *P*_min_ values can be challenging to assess in long-lived species. In chaparral systems, because of the predictable and protracted Summer/Fall dry season, it is not that difficult. The *P*_min_ that a species experiences during a typical dry season has been found to be strongly correlated to the *P*_min_ during high-intensity drought (*r* = 0.87 in ref. 21).

Xylem strength was measured in two ways. One simple estimate of tissue strength is xylem density. This was measured on the same stems used for *P*_75_ measures using Archimedes’ principle. Stems were debarked and depithed and saturated with water. The xylem was submerged in water on a four-point balance. The mass of water displaced, the temperature of the water, and the density of water were used to convert the displaced water mass to a volume. The xylem was then oven dried at 70 °C for >3 d, and the dry mass was measured. Xylem density was expressed as tissue dry mass per volume. Modulus of rupture (MOR) of stems was measured using a mechanical properties tester (Model 3342, Instron) following the methods of ref. 30. Xylem density and MOR were strongly correlated (*SI Appendix*, Fig. S1), thus we chose to use xylem density for simplicity.

Water storage of xylem (capacitance) was measured by generating pressure–volume curves on debarked and depithed samples that were about 1 cm long and 6 mm diameter. This size was necessary so samples would fit into psychrometers (Model C30, Wescor Corp.). Samples were saturated with water and weighed on a four-point balance. They were then placed into psychrometers for >2 h to allow them to equilibrate. The water potential of the psychrometers was measured with a datalogger (Model CR7, Campbell Scientific). Following equilibration, the samples were removed from the chamber, and the masses of the samples were weighed and recorded. The mean mass was taken pre- and postmeasurement and the average used to represent the mass at a water potential. Samples were then air dehydrated for 1 to 5 min and resealed in the psychrometers. This process was repeated between 8 and 16 times until the water potentials were about −6 MPa (the lower limit of these psychrometers). We used an array of 18 psychrometers and, to improve accuracy, psychrometers were calibrated with four to five salt solutions each time a species was sampled. Calibrations were done at three different cooling times, which we found was valuable to measure the most negative water potentials (the longest cooling time) and to get precise readings for more hydrated samples (shorter cooling times). To determine capacitance, curves were generated plotting relative water content (RWC; fresh weight – dry weight/saturated weight – dry weight) on the *y*-axis and in response to water potential (*SI Appendix*, Fig. S2). Capacitance was calculated as the slope (RWC/MPa) of the linear portion of the curve between about −0.3 and −1.5 MPa.

Starch content of xylem was measured using an enzymatic method for samples collected in Fall 2009. Fall was selected because this is the seasonal point when starch storage should be close to its seasonal maximum. Stems were debarked and depithed, and the remaining xylem was ground using a ball mill, consequently, only xylem starch content was measured, which was appropriate for our focus on xylem trade-offs. The starch data we used can be found in another study where our methods are fully described (36).

The proportions of difference cell types, fibers, parenchyma, and vessels were measured in cross sections of the same stems sampled for the same stems in which hydraulic traits were measured (*n* = 3 to 6 stems/species). Thin sections were made using a microtome and mounted in glycerol. Samples were examined at 200× magnification with a microscope (36).

### Structural Equation Model and Statistical Analyses.

We used an SEM approach to test our multivariate hypothesis (Fig. 1). We had two latent variables in our model: cellular trade-off and tissue strength. Cellular trade-off was represented by the area of fibers, vessels, and parenchyma measured in cross section, and tissue strength was represented by xylem density. Strength of xylem can be measured in many ways and at different scales (cell to tissue), thus it made conceptual sense to treat is as a latent variable (8); however, we did not statistically analyze it as a latent variable (*SI Appendix*, Fig. S3). Representing cellular trade-off in this way consistently led to an impossible negative error for fiber area in our models. The negative value was always very small (−0.001 to −0.006). Thus, we set fiber error to zero, which has little effect on parameter estimates when the error is very close to zero (42).

The modeling approach consisted of two parts. The first was to develop a path diagram that represented the hypothesized multivariate relationships among xylem traits (Fig. 1). In the second step, we examined if the model provided an adequate fit of the data. Prior to analysis, the data were examined in the context of parametric statistical assumptions. The data were transformed using natural log for all traits except for xylem density and water storage because the transformed relationships were less linear. The absolute value of *P*_min_ and *P*_75_ were used (*SI Appendix*, Table S2). The unstandardized coefficients that we report are transformed and scaled (*SI Appendix*, Fig. S3). All SEM tests were run using R (R version 4.0.5) package lavaan 0.6 to 8 (43).

Statisticians recommend a larger sample size than we used for SEM models that are relatively complex; however, there are reasons why we did not collect more samples. Our data set consisted of 29 species and six replicates for most variables, so we collected 174 data points for each of the nice measured factors, all of which are time consuming to measure. Another option would be to combine available data to form a larger data set, but this is not presently possible due to lack of data for the full suite of variables that we measured.

Because of our small sample size, we adjusted our model selection criteria in some ways. The goodness of fit of the SEM model was determined by a ×^2^ test that compared the fit of the model to a model with all predictor variables. The null hypothesis was that the tested model would not differ from the fully parameterized model, thus indication of a good model fit is *P* > 0.05. We report model tests from standard and Bollen–Stine bootstrapped values that are recommended for small sample sizes (3). We also report the comparative fit index and the Tucker–Lewis index (TLI), where values of >0.95 suggest good model fit. After testing our hypothesized model, we found that it fit the data reasonably well, but there were some paths in the model that were not significantly supported. We ran additional models with these paths removed. We compared these models to our initial hypothesized full model using information theoretic tests (Akaike information criterion [AIC] and Bayesian information criterion [BIC]), with an emphasis on the corrected AIC (AICc), which is adjusted for small sample size. These statistics evaluate the goodness of fit of a model and parsimony. The best-fit models have lower values of AIC and BIC, and values of >|2| are better fitting models.

In additional to analyzing raw trait values, we also ran analyses on phylogenetic independent contrasts (PICs). These were calculated for all traits using branch lengths set to 1 (Mesquite version 3.61). The same processes were followed and models run using PICs.

Additional analyses included assessing the variation across our sites for the nine traits we measured. This was done using boxplots and violin plots (R package ggplot2) and by partitioning the variance of the measured traits among species nested within each site, across the different sites, and within each species (intraspecific; R package lme4 for mixed-effect models). We also analyzed the bivariate relationships among all traits using simple Pearson correlations. We conducted a network analysis that shows correlations among traits in a correlogram. We included a strength analysis that assesses the importance of a trait in a network in the context of how strongly it is correlated with the other variables. The last analysis we conducted was a principal components analysis to describe the multivariate relationships among traits (princomp function in base R and plotted with package ggbiplot). We used a scree plot to determine that two components adequately explained the variation among our traits.

## Results

We observed large differences in trait values among the 29 shrub species we analyzed. These values spanned a large proportion of the observed variation across the globe for woody species (9, 38). Sampling many different species across different field sites leads to different sources of variation (*SI Appendix*, Table S1). We analyzed variation within sites, across sites, and intraspecifically. The general finding was that variation among traits was wide for species sampled within each site, indicating that sites were unlikely to be exerting unique effects on the measured traits (*SI Appendix*, Fig. S4). This is also supported by the large proportion of overall variance contributed by species nested within site (*SI Appendix*, Fig. S5). One exception was for *P*_min_ and *P*_75_, which at one site (Phantom site) did not have species with values as extremely negative as found at the other sites (*SI Appendix*, Fig. S4); however, this likely occurred because we did not randomly sample species within a site and instead chose unique species. The Phantom site was established last, and the site contained species that experience highly negative *P*_min_ values, but we elected to not sample them because they were already in our data set from other sites. Moreover, this site receives the second-lowest average rainfall among the four sampled, and it also experiences hot temperatures, suggesting it is not a mesic site in our study (see ref. 36).

**Fig. 2.**
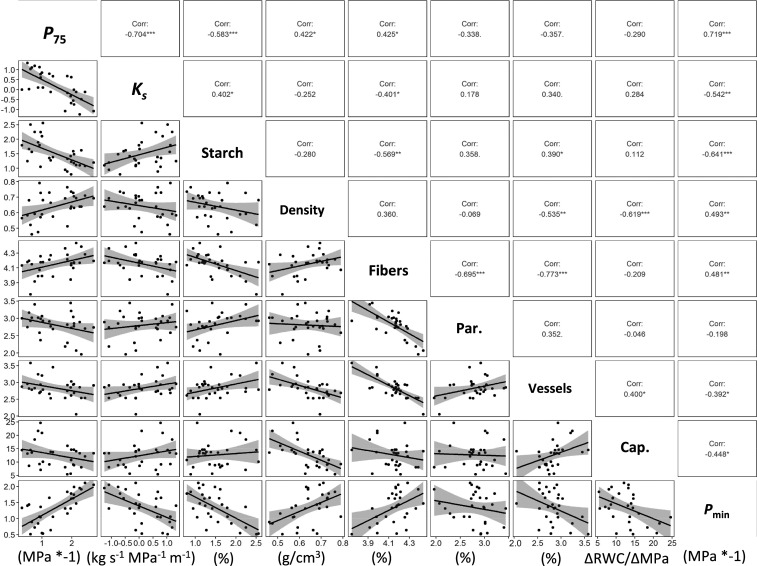
Bivariate correlations among all the traits with associated r-values and significance (*** < 0.001; ** < 0.01, * < 0.05, . < 0.10) for raw trait values, and those for PICs are in the supplemental figures (*SI Appendix*, Fig. S7). Cap. refers to capacitance or water storage, and Par. is short for parenchyma. *P*_75_ represents the water potential at 75% loss of hydraulic conductivity and estimates embolism resistance, and *K*_s_ is xylem specific conductivity and represents transport efficiency. Details about other traits are described in *Materials and Methods*.

Significant and strong bivariate correlations were observed among many of the measured traits (Fig. 2). The extremes were *P*_min_, which was significantly correlated with all variables except parenchyma area, and parenchyma area, which was only correlated with one other trait (Fig. 2). Not only was *P*_min_ correlated to most variables, it also had many strong associations (*SI Appendix*, Fig. S6). Fiber area was another trait with many significant and strong associations with other traits (Fig. 2 and *SI Appendix*, Fig. S6). Making these same comparisons with PICs generally showed the same patterns (*SI Appendix*, Fig. S7).

Summarizing the multivariate relationships among these traits using principal component (PC) analysis showed clear patterns where PC1 captured the inverse relationships between safety and efficiency, tissue strength and starch storage, and vessels and fibers (*SI Appendix*, Fig. S8). PC2 described the inverse relationship between water storage and xylem density and parenchyma and fibers. The same patterns were apparent when analyzed using PICs (*SI Appendix*, Fig. S8).

**Fig. 3.**
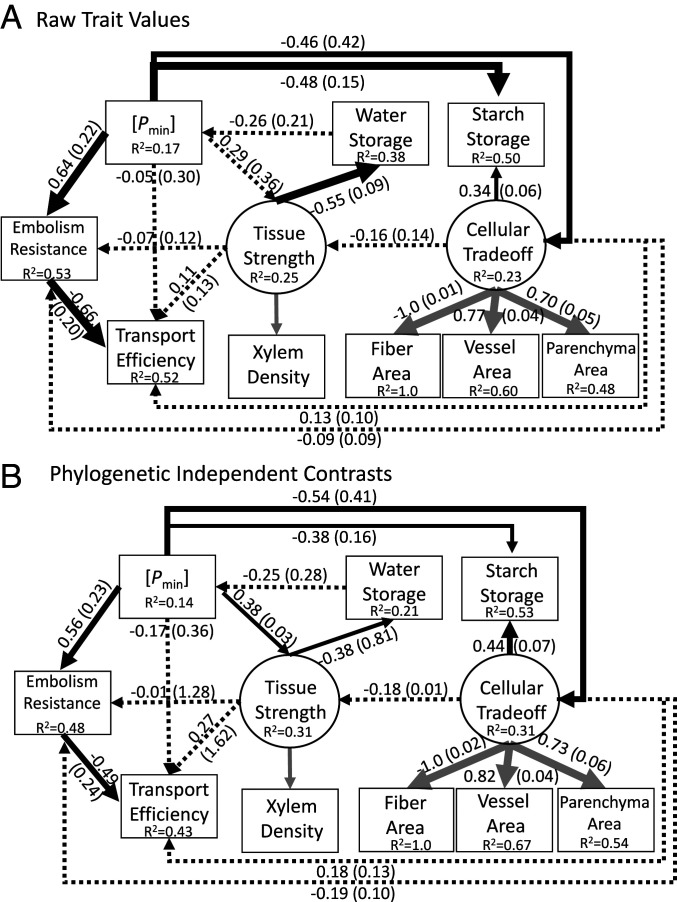
Results from our analyzed SEM model for raw trait values (*A*) and PICs (*B*). The weights of the solid arrows correspond to *P* values where the thickest is <0.001, intermediate <0.01, and thinnest is <0.05. The dotted arrows correspond to *P* > 0.05. The values shown along paths are standardized coefficients and SEs in parentheses (*SI Appendix*, Fig. S3 shows unstandardized coefficients). The variance explained (R^2^) is shown for each trait. Latent variables are connected to their measured traits by gray arrows. Values are not shown for xylem density because this trait is included within the “tissue strength” variable, and the values there apply to xylem density.

The analyzed SEM model produces different types of variables and coefficients. The coefficients shown along the paths (predictors) represent the relationship between variables (Fig. 3). They are standardized and represent the change expected (positive or negative) if a predictor variable is varied by one SD. In cases where there are multiple predictors for a single trait (embolism resistance, transport efficiency, tissue strength, and starch storage), the coefficients represent partial regression coefficients. We include both standardized coefficients (Fig. 3) and unstandardized coefficients in the transformed units of the measured traits (*SI Appendix*, Fig. S3 and Table S2).

**Fig. 4.**
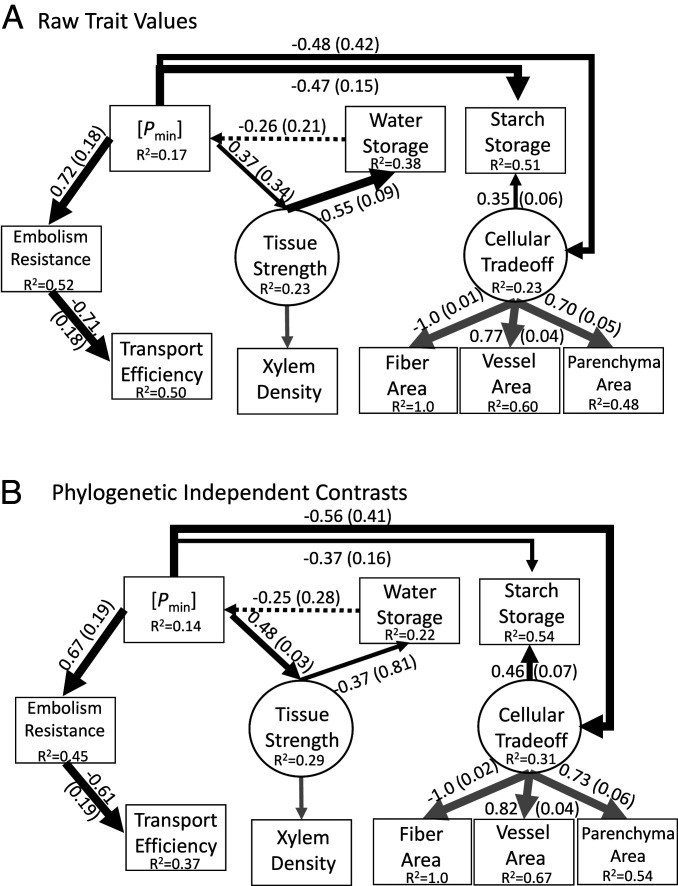
The best-fitting SEM models for raw trait values (*A*) and PICs (*B*). The weights of the solid arrows correspond to *P* values where the thickest is <0.001, intermediate <0.01, and thinnest is <0.05. The dotted arrows correspond to *P* = 0.198 (*A*) and 0.220 (*B*). The values shown along paths are standardized coefficients and SEs in parentheses (*SI Appendix*, Fig. S3 shows unstandardized coefficients). The variance explained (R^2^) is shown for each trait. Latent variables are connected to their measured traits by gray arrows. Values are not shown for xylem density because this trait is included within the “tissue strength” variable, and the values there apply to xylem density.

The overall hypothesized model (Fig. 1) was a good fit of the data [i.e., the fit was not significantly different from a saturated model where all the possible paths were included (Fig. 3*A* and *SI Appendix*, Fig. S3 and Table 1)]. The same was true for the model using PICs (Fig. 3*B* and *SI Appendix*, Fig. S3 and Table 1). Although the model provided adequate support for covariation among the traits, there were six paths in the model that had high *P* values (Fig. 3 and Table 1); moreover, the TLI was <0.95. To investigate, we created models with these paths removed and compared the effect on model fit (Table 1). For the six paths with large *P* values, we proceeded by removing variables with the largest *P* values, rerunning the model, and evaluating the effect on the *P* values and model fit. In all cases, removing the paths had little effect on the large *P* values and model fit, so we removed them all (Table 1). We found that the best-fit model was the full model minus six paths with high *P* values (Fig. 4 and Table 1). The path between water storage and *P*_min_ was also not significant (*P* = 0.198); however, removing this path led to a poorer-fitting model (Table 1).

**Table 1.** Model fit statistics comparing the fit of different models to our hypothesized model (full model, Fig. 2)

	K	AICc*	AIC	BIC	CFI^†^	TLI^†^	LL	df	*×* ^2^	P	P^‡^
Model raw traits											
1. All paths *P* > 0.380^§^	20	320.16	215.16	242.50	0.970	0.957	−87.58	25	28.88	0.269	0.548
2. Cap.→*P*_min_ + *P* > 0.380	21	348.03	216.02	244.74	0.971	0.957	−87.01	24	27.75	0.274	0.568
3. Full	26	926.42	224.42	259.97	0.945	0.897	−86.21	19	26.14	0.126	0.446
Model PICS											
1. All paths *P* > 0.330^§^	20	237.60	117.60	144.24	0.961	0.944	−38.80	25	29.93	0.227	0.506
2. Cap.→*P*_min_ + *P* > 0.330	21	272.44	118.44	146.42	0.962	0.944	−38.22	24	28.76	0.229	0.525
3. Full	26	1,527.9	123.86	158.49	0.959	0.922	−35.93	19	24.19	0.189	0.511

* This is adjusted for small sample size.

^†^ The comparative fit index (CFI) and Tucker–Lewis index indicate good model fits if values are ≥0.95.

^‡^ These *P* values are from Bollen–Stine bootstrapping.

^§^ The six paths removed were between efficiency and cellular trade-off, minimum water potential, and strength and between embolism resistance and cellular trade-off and strength and between strength and cellular trade-off.

Among the relationships that were predicted based on our hypotheses, many were not supported by the model. The model showed that transport, tissue strength, and starch storage functions were independent of one another. An important result is that a cellular trade-off was associated with *P*_min_ and was independent of tissue strength. This trade-off was directly linked to starch storage, but it was not associated with any other traits. A network analysis shows *P*_min_ to be a “hub” trait due to the number and strength of the associations (*SI Appendix*, Fig. S6). Another direct predicted relationship supported was the inverse relationship (trade-off) between safety from embolism and efficiency.

The results for the raw traits and PICs were virtually identical, thus we focus on raw trait values for simplicity. The direct relationships that were not supported were those between *P*_min_ and efficiency, tissue strength and efficiency and embolism resistance, cellular trade-off and embolism resistance, efficiency, and tissue strength. *P*_min_ and efficiency were associated through a shared relationship with embolism resistance. Tissue strength was not directly related to either embolism resistance or efficiency, thus any relationship it has with these traits is through *P*_min_ and possibly water storage. These indirect relationships highlight *P*_min_ as a central parameter underlying xylem trait relationships.

To explore the influence of *P*_min_ on trait relationships further, we created models with *P*_min_ removed (*SI Appendix*, Figs. S9–S11). The best-fit model was produced from three candidate models (*SI Appendix*, Table S3). An important result is that cellular trade-offs take on a central role, directly or indirectly affecting all other traits when *P*_min_ is removed (*SI Appendix*, Figs. S9–S11). A good example of how *P*_min_ is exerting influence is between cellular trade-off and tissue strength, both of which have direct paths from *P*_min_ (Fig. 4). In the full model with *P*_min_ this path is insignificant, and the partial standardized regression coefficient is −0.16 (Fig. 3*A*), thus for every SD increase in cellular trade-off, there is a −0.16 decline in tissue strength (a result of the inverse relationship between vessel area and xylem density). In the model without *P*_min_, the coefficient goes to −0.47 and it is significant, a result almost entirely due to the absence of *P*_min_. This analysis did not support a direct association between tissue strength and embolism resistance (*SI Appendix*, Table S3 and Figs. S9–S11).

## Discussion

We proposed a multivariate hypothesis regarding trade-offs in xylem function that predicted how key functional traits were interrelated. These trade-offs have been mostly evaluated individually (5, 6, 8, 37); however, none have done so as part of a multivariate testable model. Such models allow for the identification of direct and indirect relationships, as well as the dependence of traits on one another. Results using PICs were the same as those with raw trait values, suggesting that shared ancestry cannot explain the associations among our sampled traits.

We found that the xylem functions (transport, strength, water storage, and carbohydrate storage) were independent of one another, and the only trait linked to all of them was *P*_min_. Bivariate relationships indicated significant associations between tissue strength and embolism resistance and cellular trade-offs, but these were not supported by our final SEM model. A key reason for this result is the presence of *P*_min_ in the model and its strong associations with nearly all traits. To explore this, we created a model with *P*_min_ removed. In this model, a cellular trade-off was found to directly affect embolism resistance, tissue strength, and starch storage and indirectly affect efficiency and water storage (all traits in the model). This supports one of our main hypotheses that the balance between the different cell types is a central structural factor affecting xylem function; moreover, it suggests that the cellular functional divisions and the range of different cellular sizes, shapes, and wall thicknesses cannot fully overcome trade-offs (6, 31).

Taken as a whole, the effect of cellular trade-offs and tissue strength is not independent of *P*_min_. The relationships between embolism resistance and tissue strength and cellular trade-offs are hypothesized to occur because of the need to reinforce vessels against implosion (29, 30), which is more of a threat in species that experience more negative hydrostatic pressures and that are highly resistant to embolism. Thus, the hypothesis that predicts these relationships also predicts a lack of independence among these traits, as we found. One aspect of cellular strength not included here is direct estimate of implosion resistance of individual vessels or vessel pairs (29), which if independent of bulk tissue strength, could affect model results.

Our results highlight the central importance of *P*_min_ as an explanatory variable (11). In the context of a trait network, *P*_min_ is a “hub” trait (1). *P*_min_ represents the level of dehydration a plant experiences, and within a similar environment and measured at midday, it integrates many plant traits such as rooting patterns (44), stomatal responses (11, 45), leaf turgor and hydraulic conductance (11), and hydraulic conductance of the plant and soil system. The hub effect of *P*_min_ in our model of xylem traits likely occurs because it captures variability in many fundamental aspects of plant function that are associated with xylem function in an example of concerted convergence. A strong relationship between *P*_min_ and embolism resistance is well established (20), but our study shows that tissue strength and embolism resistance and cellular trade-offs are not related independent of *P*_min_ and that *P*_min_ is linked to cellular trade-offs.

The association between *P*_min_ and cellular trade-offs may arise for structural and storage reasons. A shift to containing less fibers and more parenchyma may destabilize the xylem, creating a risk of vessel implosion (30). If so, then more negative *P*_min_ would be associated with more fiber area and reduced parenchyma and vessel area, which was supported as seen among bivariate correlations [note, parenchyma is not significant; ref. 46]. These ideas suggest a link between cellular trade-offs and tissue strength, a relationship not independent of *P*_min_. Shifting from less fibers to more parenchyma is also associated with greater starch storage (6, 8, 35), and starch storage is strongly associated with *P*_min_ (36). Expressing cellular trade-offs as a latent variable described by all cell types helped to identify important relationships; however, parenchyma performs important functions beyond storage such as defense, radial transport, and refilling of tracheary elements, and these additional functions warrant further study (12, 31, 47). Different types of parenchyma cells and arrangements (axial, ray, paratracheal, contact, isolation, etc.) may associate differently with different functions and predictors (12, 31), which is likely due to functional differences among these parenchyma types (8).

Storage of xylem starch and carbohydrates is an important trait related to drought tolerance and growth (48) and plays a role in xylem refilling (49). We hypothesized that *P*_min_ drives starch storage because starch is hydrolyzed to osmoregulate in dehydration tolerant species that experience highly negative *P*_min_ (38, 50), and this was consistent with our data. The connection between starch storage and *P*_min_ may drive the association between starch storage and embolism resistance (36). Understanding the dynamics of carbohydrates, including its transport, is an important area of active research (34, 50).

Water storage is the only trait in our model that affects *P*_min_, which gives it the potential to play a critical role in overall xylem function (50). Water storage indirectly links tissue strength to the transport functions through *P*_min_. Xylem density (tissue strength) correlates with many different xylem traits and ecological and life history traits (51), and its effects on water storage and *P*_min_ are likely important in this context. One caveat is that the association between water storage and *P*_min_ was in the best-fitting model, but it was not strongly supported (*P* > 0.05 for the path connecting water storage to *P*_min_). This is mainly due to the hypothesized complex relationship between *P*_min_, tissue strength, and water storage. This relationship is modeled as nonrecursive (a loop) where water storage indirectly affects itself through its effect on *P*_min_, which in turn affects tissue strength, then back to water storage. Feedback loops are likely important in the context of selection for and relationships among traits affecting *P*_min_ and are an important area for further study.

Other well-supported relationships in our model are the link between *P*_min_ and embolism resistance and the trade-off between safety from embolism and efficiency. Species widely differ in the *P*_min_ they experience, and *P*_min_ is correlated to drought resistance and embolism resistance (18, 52). This is consistent with the hypothesis that embolism resistance is an important trait associated with plant dehydration avoidance/tolerance strategy. Our results are also consistent with the well-studied trade-off between safety from embolism and efficiency (9). At the global scale, this relationship is weak (23), and it has been argued that the multiple traits affecting this trade-off over diverse selective environments has uncoupled these traits (16). Our study is in a semiarid ecosystem, where strong water limitation likely constrains the range of responses.

Hydraulic efficiency was only strongly and significantly associated with embolism resistance. Bivariate relationships showed some significant relationships, including an association with *P*_min_ and cellular trade-offs (fiber area), yet none of our models suggested direct associations with hydraulic efficiency. Efficiency is indirectly associated with *P*_min_ through a direct path between *P*_min_ and embolism resistance, and in models without *P*_min_, it is similarly indirectly associated with cellular trade-offs. *P*_min_ could directly affect efficiency if expansion of large vessels was limited by turgor pressure, especially if wider vessels take longer to develop (27); however, chaparral shrubs do not have very large vessels globally speaking (53), thus during a typical hydrological year, interspecific differences may be unlikely. Nevertheless, during a drought, there will certainly be a reduction in xylem growth increment and vessel diameter, which may be driven by *P*_min_.

We also did not find a direct connection between tissue strength and transport efficiency. This path was predicted to arise because of the need for denser tissues in response to *P*_min_. The denser tissues were hypothesized to compromise efficiency between vessels with narrower diameters and thicker walls because thicker walls increase the path length through the pits where sap flows in between vessels, and this would decrease hydraulic efficiency (24). Tissue strength is driven by fiber traits (fiber abundance and wall thickness), so angiosperms can adjust their tissue strength independent of transport. However, these relationships may manifest in lineages where the developmental connection between fiber and vessel walls is strong (24).

The ecological context for our study is likely important to understand relationships with transport efficiency. Efficient transport of xylem is broadly associated with fast acquisition and use of resources, competitive ability, and has been linked to greater photosynthetic capacity and may lead to lower construction costs of stems (23). Our study focused on shrubs in a semiarid ecosystem where xylem efficiency may be unlikely to be the primary trait affecting fitness. By contrast, limited water is a likely a primary selective force for traits associated with drought survival. As such, embolism resistance may be under stronger selection than xylem efficiency (18, 52). In ecosystems with greater resources and dominated by trees, the arrows between efficiency to other traits may reverse, whereby it becomes a predictor instead of a response variable. We tried this in the present study, and when we reversed the path between efficiency and embolism resistance in the best-fit model (Table 1), the resulting model fit was poor (χ^2^ = 37.77, df = 25, *P* = 0.049). Direct manipulative tests to examine questions about adaptive significance of xylem traits is an area where more research is needed.

Hypotheses underpinning trait relationships with starch storage may change when carbon gain is limited over a long period by an unfavorable environment. It is well documented that when plants are carbohydrate limited, they produce less-dense tissues (54). Thus, when carbon gain is marginal relative to carbon expenses and phloem transport is impaired (55), starch availability to cambia may be limited and drive reduced tissue density and strength (56, 57). Under such conditions, a direct link between tissue strength and embolism resistance may be important as mechanically weak vessels become vulnerable to collapse (54, 56).

We conclude that xylem traits are broadly governed by trade-offs among cellular traits related to transport, mechanical support, and storage and that the *P*_min_ experienced by plants in the field exerts a strong influence over these relationships. While angiosperms have evolved different cell types that have different functions within the xylem, and there are important functional trade-offs associated with the relative proportions of these different cell types. The important effects of *P*_min_ on xylem traits likely arises because it places a direct mechanical strain on tissues that requires reinforcement to avoid cellular implosion; nevertheless, *P*_min_ can affect xylem function by other pathways and traits not considered in our model because it integrates many functional attributes of plants.

## Supplementary Material

Supplementary File

## Data Availability

All study data are included in the article and/or *SI Appendix*. Previously published data were used for this work [some data were previously published in a very different format in Pratt et al. (36)].
