# Supplementary material for: Trade-offs among transport, support, and storage in xylem from shrubs in a semiarid chaparral environment tested with structural equation modeling
Source: Proc Natl Acad Sci U S A. 2021 Aug 13;118(33):e2104336118. doi: 10.1073/pnas.2104336118 (PMC8379947; doi:10.1073/pnas.2104336118)
Supplement: Supplementary File [file pnas.2104336118.sapp.pdf]

Supplementary Information for

Tradeoffs among transport, support, and storage in xylem from shrubs in a semi-arid chaparral environment tested with structural equation modeling

Pratt, R.B.
Jacobsen, A.L.
Percolla, M.I.
De Guzman, M.E.
Traugh, C.A.
Tobin, M.F.

Correspondence: R. Brandon Pratt
Email: rpratt@csub.edu

This PDF file includes:

- Tables S1 to S3
- Figures S1 to S11
- SI References

Table S1. List of species and families included in the present study (more information in Pratt *et al*. 2021).

Species	Family
Adenostoma fasciculatum	Rosaceae
Adenostoma sparsifolium	Rosaceae
Arctostaphylos glandulosa	Ericaceae
Arctostaphylos glauca	Ericaceae
Bernardia incana	Euphorbiaceae
Ceanothus crassifolius var. *crassifolius*	Rhamnaceae
Ceanothus spinosus	Rhamnaceae
Ceanothus vestitus	Rhamnaceae
Cercocarpus betuloides	Rosaceae
Frangula californica	Rhamnaceae
Fraxinus dipetala	Oleaceae
Fremontodendron californicum	Malvaceae
Heteromeles arbutifolia	Rosaceae
Juglans californica	Juglandaceae
Keckiella ternata	Plantaginaceae
Malosma laurina	Anacardiaceae
Prunus emarginata	Rosaceae
Prunus fasciculata var. *fasciculata*	Rosaceae
Purshia tridentata	Rosaceae
Quercus berberidifolia	Fagaceae
Quercus cornelius-mulleri	Fagaceae
Quercus wislizeni	Fagaceae
Rhamnus ilicifolia	Rhamnaceae
Rhus aromatica	Anacardiaceae
Rhus ovata	Anacardiaceae
Ribes malvaceum	Grossulariaceae
Ribes speciosum	Groassulariaceae
Sambucus nigra ssp. caerulea	Adoxaceae
Senegalia greggii	Fabaceae

Table S2. All the mean values used in the manuscript, including how they were transformed and scaled for modeling purposes in the notes below. Fiber, vessel, and parenchyma (Par.) are the percentages of each cell type in cross section.

Species	P_{75} (MPa)	K_s	Starch (%)	Xylem Density (g/cm^3)	Fiber (%)	Vessel (%)	Par. (%)	P_{min} (MPa)	Water Storage
Adenostoma_fasciculatum	-7.22	0.50	2.21	0.74	62.50	13.60	24.80	-5.91	10.75
Adenostoma_sparsifolium	-7.13	0.72	4.12	0.63	51.45	17.26	31.28	-3.72	5.63
Arctostaphylos_glandulosa	-7.98	0.47	4.41	0.63	64.50	14.68	17.30	-7.27	8.79
Arctostaphylos_glauca	-10.21	0.62	5.04	0.64	80.28	12.61	7.11	-6.77	8.04
Bernardia_myricifolia	-15.36	0.34	3.30	0.69	66.20	18.33	15.46	-7.80	13.30
Ceanothus_crassifolius	-11.51	0.89	2.93	0.71	72.41	12.64	14.95	-8.25	12.12
Ceanothus_spinosus	-7.27	1.86	3.40	0.64	64.03	20.67	15.30	-5.06	14.62
Ceanothus_vestitus	-9.64	0.29	3.00	0.64	64.61	17.38	18.01	-7.02	13.28
Cercocarpus_betuloides	-6.37	1.96	2.43	0.76	67.62	15.61	16.75	-6.25	5.42
Frangula_californica	-2.38	1.10	6.62	0.48	54.00	25.57	20.43	-2.34	21.63
Fraxinus_dipetala	-1.66	3.79	3.47	0.58	65.21	19.24	15.55	-4.07	13.16
Fremontodendron_californicum	-2.72	2.34	8.20	0.59	54.06	24.59	21.35	-1.72	20.88
Heteromeles_arbutifolia	-8.58	0.89	3.03	0.69	84.30	7.78	7.92	-4.36	14.03
Juglans_californica	-2.13	3.29	9.48	0.59	50.21	16.87	30.11	-2.07	18.39
Keckiella_ternata	-6.38	0.84	3.75	0.51	72.00	18.51	9.89	-3.18	16.07
Malosma_laurina	-1.93	3.03	4.77	0.46	63.37	16.16	20.46	-2.45	13.49
Prunus_emarginata	-3.92	1.95	4.00	0.58	53.62	31.61	14.78	-2.88	13.68
Prunus_fasciculata	-7.76	0.57	4.89	0.73	60.28	18.77	20.96	-5.11	9.08
Purshia_tridentata	-5.91	0.34	2.58	0.69	78.41	12.66	8.93	-3.73	8.05
Quercus_berberidifolia	-3.49	2.21	3.86	0.73	67.35	14.29	18.36	-5.19	9.11
Quercus_cornelius-mulleri	-2.59	1.83	9.42	0.73	64.98	15.21	19.81	-3.90	7.08
Quercus_wislizeni	-1.75	1.02	5.25	0.68	66.40	16.71	23.67	-4.21	8.60
Rhamnus_ilicifolia	-7.94	0.99	3.24	0.71	64.91	19.08	16.01	-5.71	14.61
Rhus_aromatica	-2.59	2.17	2.85	0.57	70.55	18.78	10.67	-2.83	24.61
Rhus_ovata	-1.38	1.00	5.98	0.56	62.25	17.69	20.05	-3.80	14.48
Ribes_malvaceum	-3.62	0.88	3.58	0.66	71.54	12.53	15.93	-1.89	13.82
Ribes_speciosum	-2.80	1.11	12.90	0.68	65.16	16.15	18.69	-1.70	10.10
Sambucus_nigra	-1.84	2.81	12.27	0.52	42.22	35.78	18.65	-1.67	14.54
Senegalia_greggii	-2.50	3.10	6.01	0.79	58.77	14.66	13.20	-2.89	9.20

Notes: See Figure 2 in main manuscript for units. For SEM analyses, the absolute value of P_{75} and P_{min} were used. All data were natural log transformed except for xylem density, and water storage. To similarly scale data in the SEM model, water storage was divided by 10, and xylem density was multiplied by 10.

Table S3. Model fit statistics comparing the fit of different models that have P_{min} removed from our hypothesized model (full model, Figures S9, S10, S11).

Model Raw Traits	K	AICc[1]	AIC	BIC	CFI[2]	TLI[2]	LL	df	X^2	P	P[3]
1. -P75→Strength+ P>0.320	14	226.02	196.01	215.16	0.949	0.924	-84.01	14	18.09	0.203	0.303
2. -All paths P>0.320[4]	15	233.28	196.35	216.86	0.958	0.932	-83.18	13	16.43	0.227	0.309
3. Full	17	254.87	199.23	222.47	0.947	0.899	-82.62	11	13.30	0.169	0.240
Model PICs	K[5]	AICc[1]	AIC	BIC	CFI[2]	TLI[2]	LL	df	X^2	P	P[3]
1. -P75→Strength+ P>0.320	13	220.85	196.58	214.35	0.930	0.902	-85.29	15	20.65	0.148	0.265
2. -All paths P>0.320[4]	14	225.49	195.48	214.62	0.956	0.934	-83.74	14	17.56	0.227	0.333
3. Full	16	243.73	198.40	220.27	0.945	0.903	-83.20	12	16.47	0.170	0.308

1. This is adjusted for small sample size.
2. The comparative fit index (CFI) and Tucker-Lewis index indicate good model fits if values are ≥0.95.
3. These P-values are from Bollen-Stine bootstrapping.
4. The two paths removed were between efficiency and cellular tradeoff, and strength.
5. These values differ from the raw traits because fiber area error was set to zero to avoid negative standard errors for PICs only.

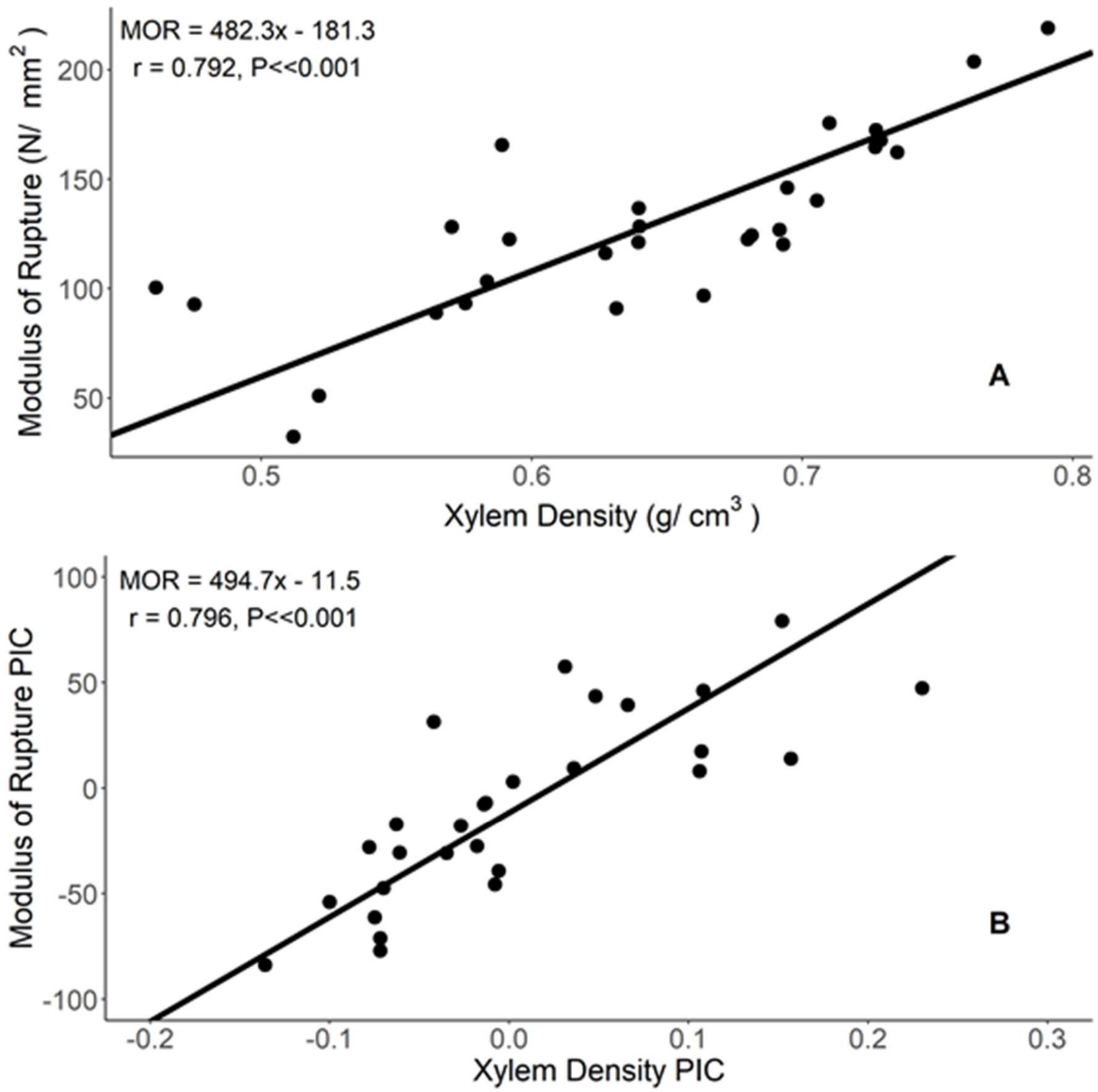

Figure S1. Plot showing that tissue strength is strongly associated with xylem density. Equations are for standardized major axis regression as are the Pearson r-values and P-values. The lower panel is the same relationship for phylogenetically independent contrasts (PICs).

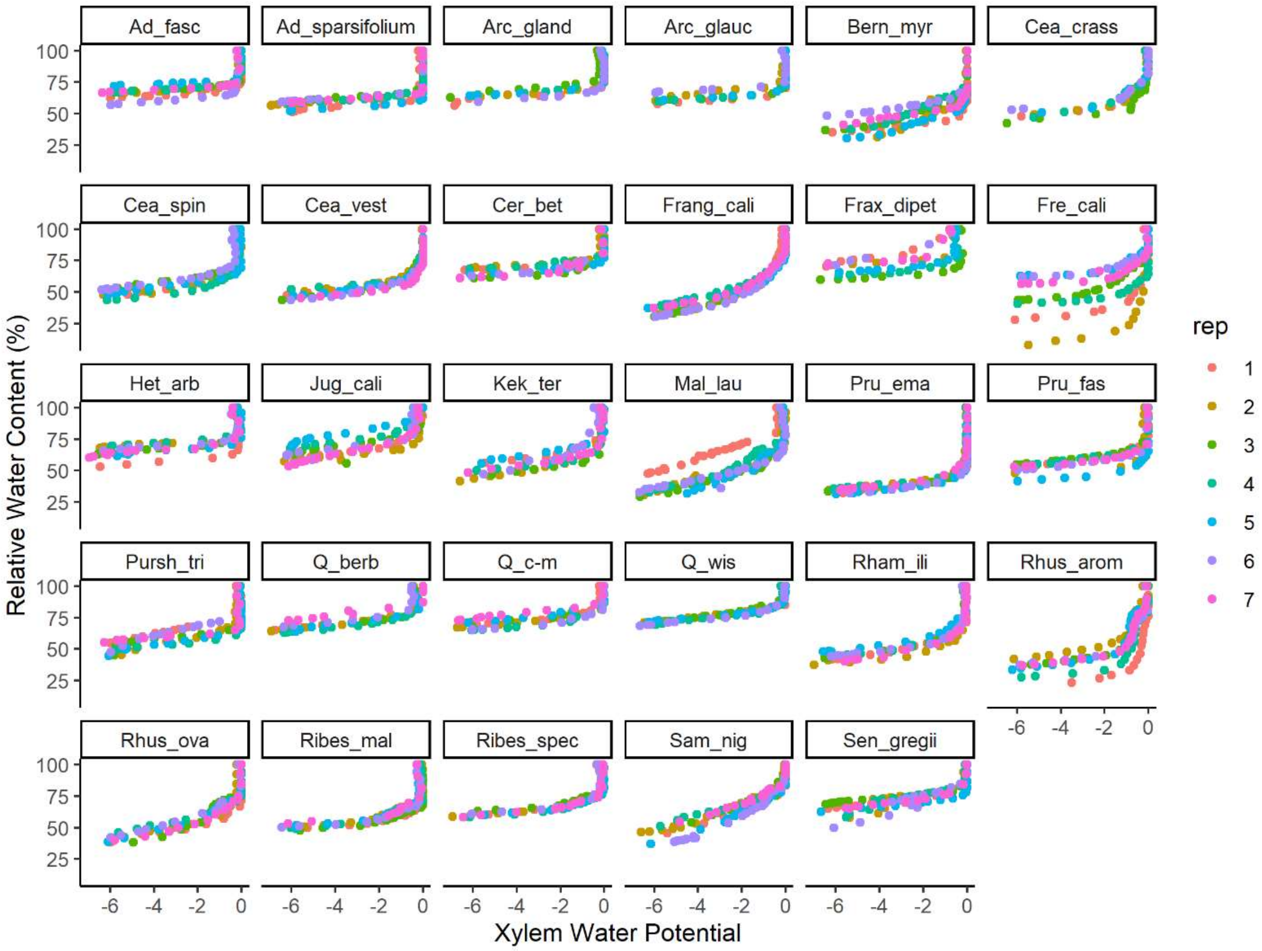

Figure S2. Individual pressure volume curves used to quantify capacitance from stems. Species names are shortened from Table S2. Rep refers to independent replicates (different stems from different individuals, n=6 or 7). For capacitance estimates, we used the linear portion of the curve between a water potential range of about -0.3 to -1.5 MPa. This range occurred after the initial steep drop in relative water content when water potential did not change and before the curve flattened out after about -2 MPa (see Pratt and Jacobsen 2017).

Full Initial Model

Raw Traits

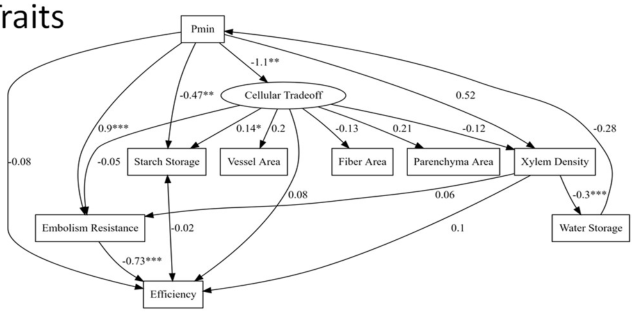

PICs

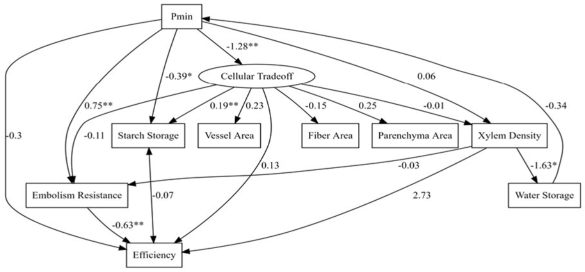

Final Model

Raw Traits

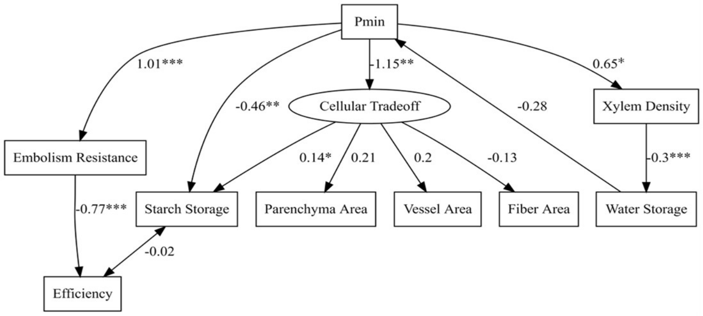

PICs

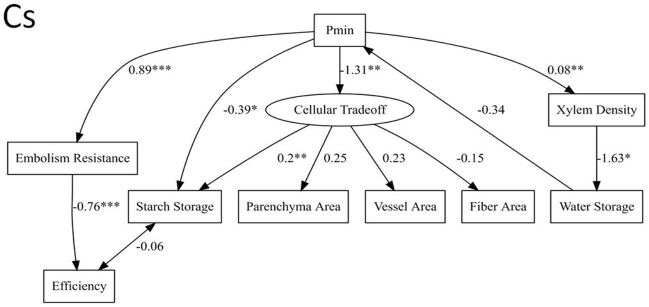

Figure S3. Results from our analyzed structural equation models for raw trait values and phylogenetic independent contrasts (PICs) for the full initial model outlined in Figures 1 and 3, and the final model shown in Figure 4. Coefficients are unstandardized (data in Table S2) and asterisks denote significance among paths (***P<0.001, **P<0.01, *P<0.05). Cellular tradeoff is a latent variable described by areas of different cell types (P-values < 0.001 for all cell areas).

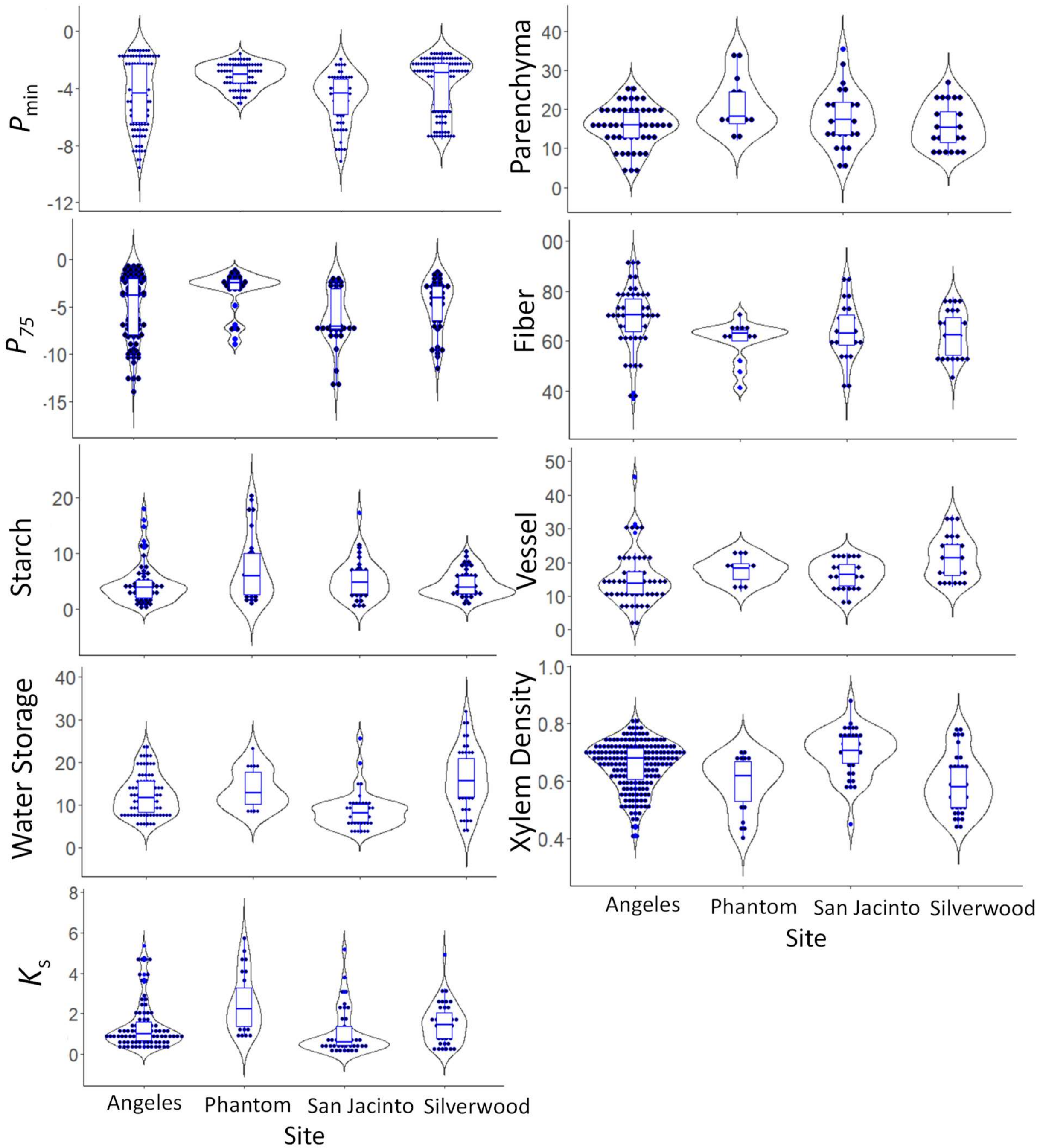

Figure S4. Boxplots and dotplots within violin plots for the nine measured traits showing the distribution of the measured variables across the four sites (field site locations and meteorological information is in Pratt *et al*. 2021). See Figure 2 for units.

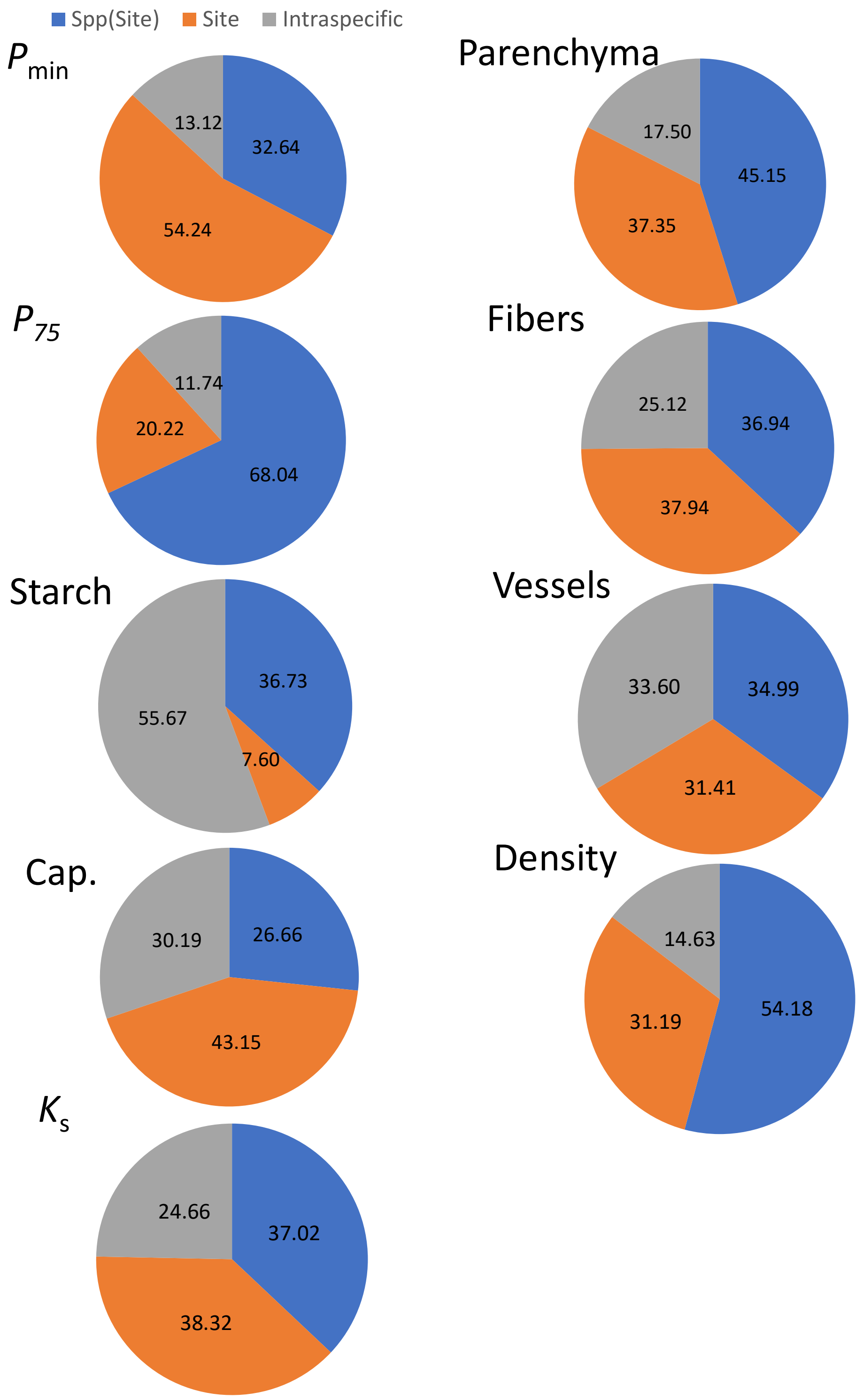

Figure S5. The percentage of variance for each measured trait partitioned by species nested within each site, across sites, and within each species (intraspecific). Cap. refers to capacitance or water storage.

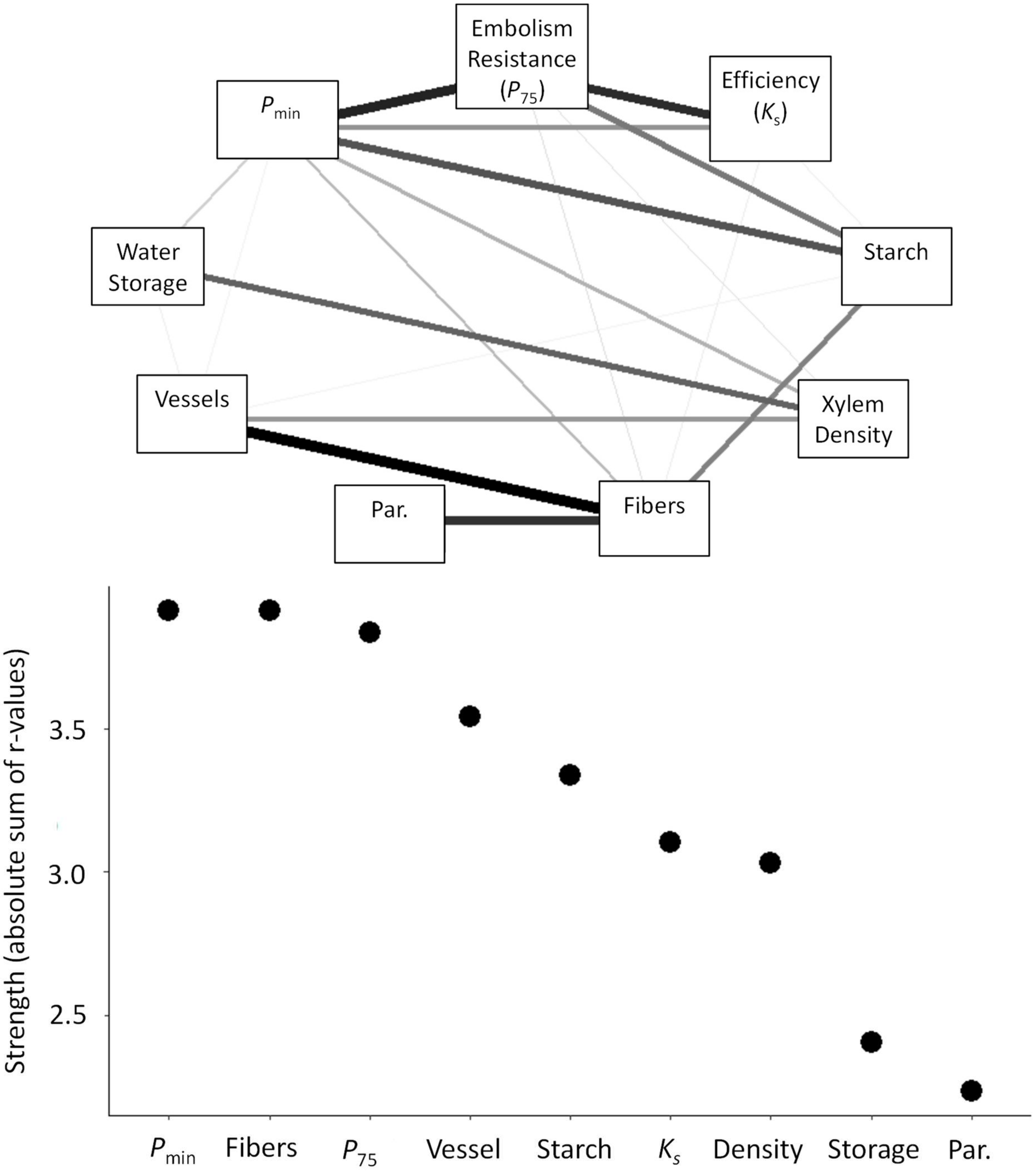

Figure S6. Network relationships among traits and strength of traits. The thickest lines in top panel have a P<0.001 and the thinnest ones are P<0.05, while ones of intermediate thickness are P<0.01). Traits are ordered by their strength, which describes their importance in the context of the overall strength of the associations. P_{min} is supported as a “hub” trait.

Phylogenetic Independent Contrasts

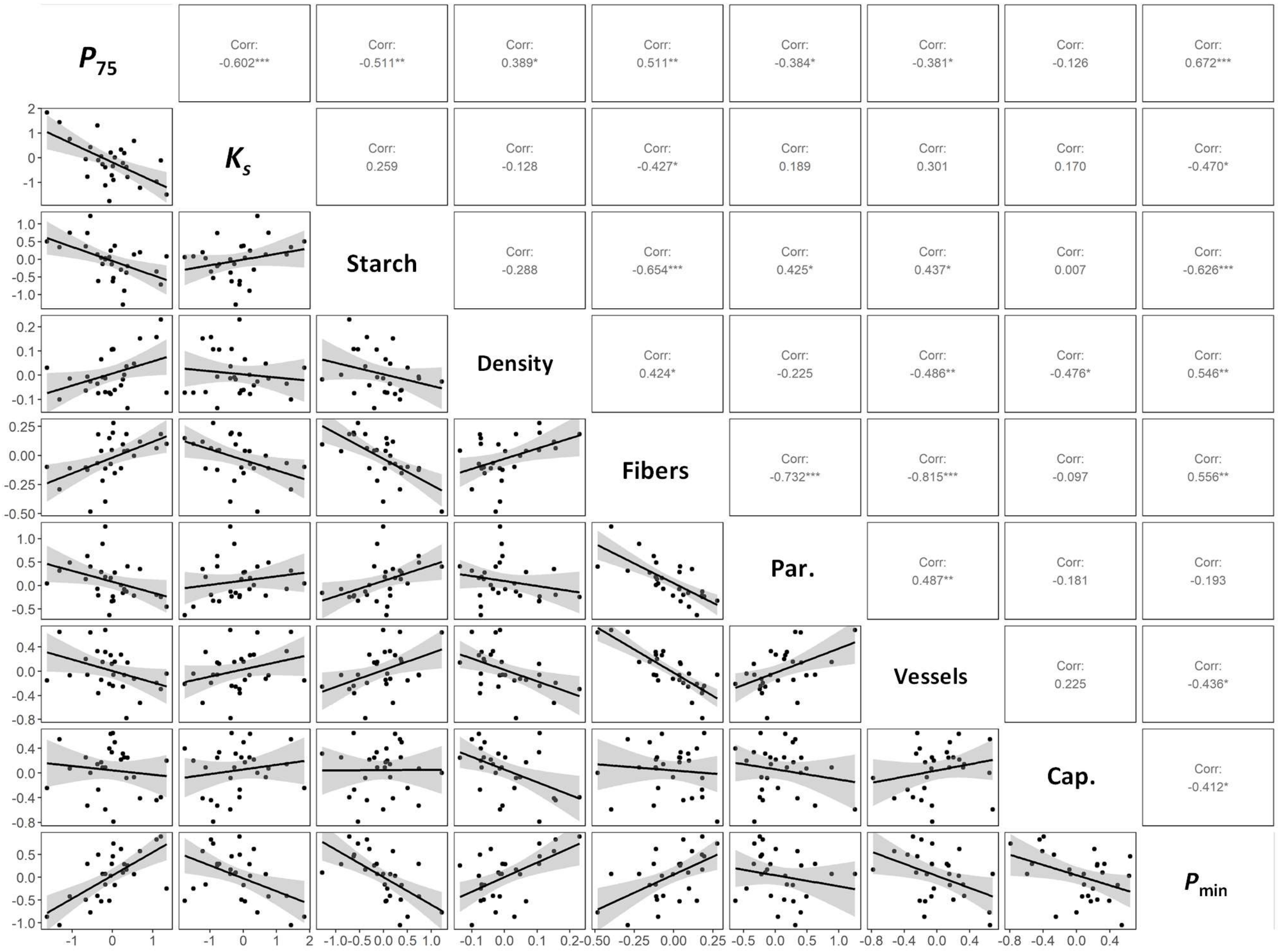

Figure S7. Bivariate correlations among all the traits with associated r-values and significance (***<0.001; **<0.01, *<0.05) for phylogenetic independent contrasts.

Raw Trait Values

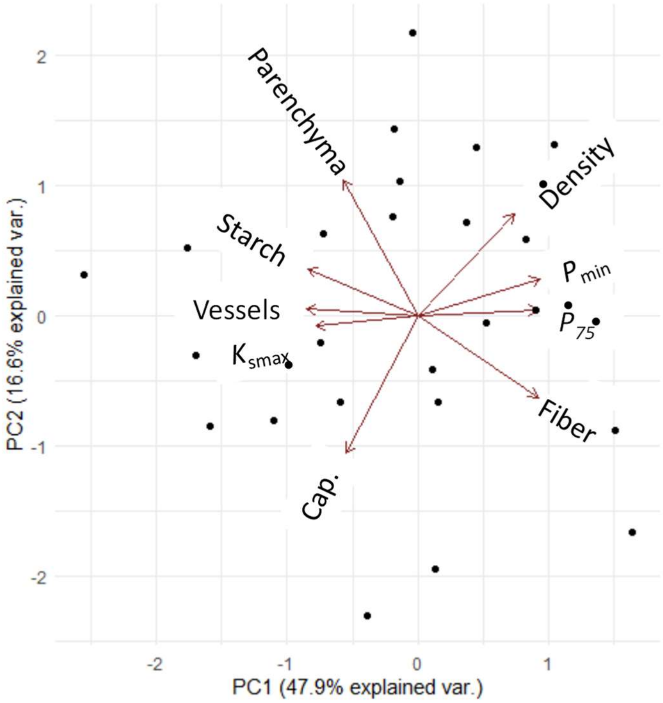

Phylogenetic Independent Contrasts

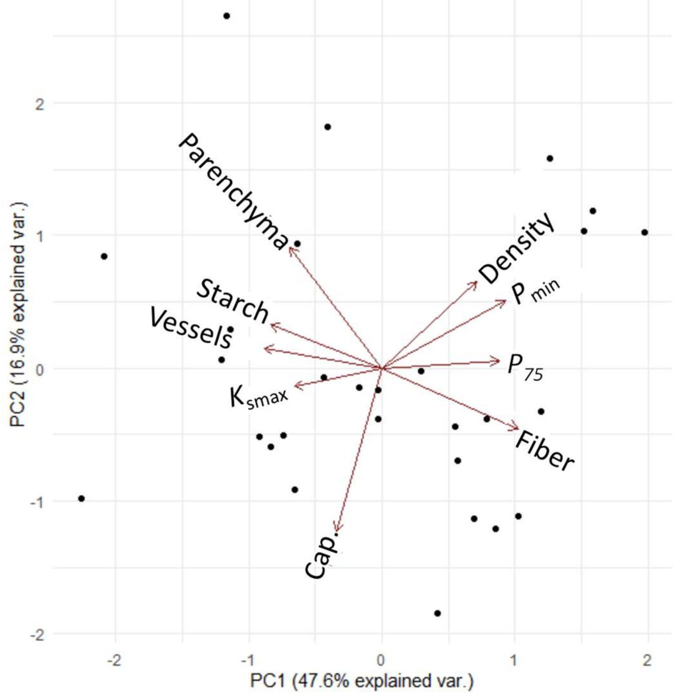

Figure S8. Principal components analysis for the first two components of the analyzed traits. The top panel is for the raw and transformed trait values and the lower panel is for phylogenetic independent contrasts (PICs).

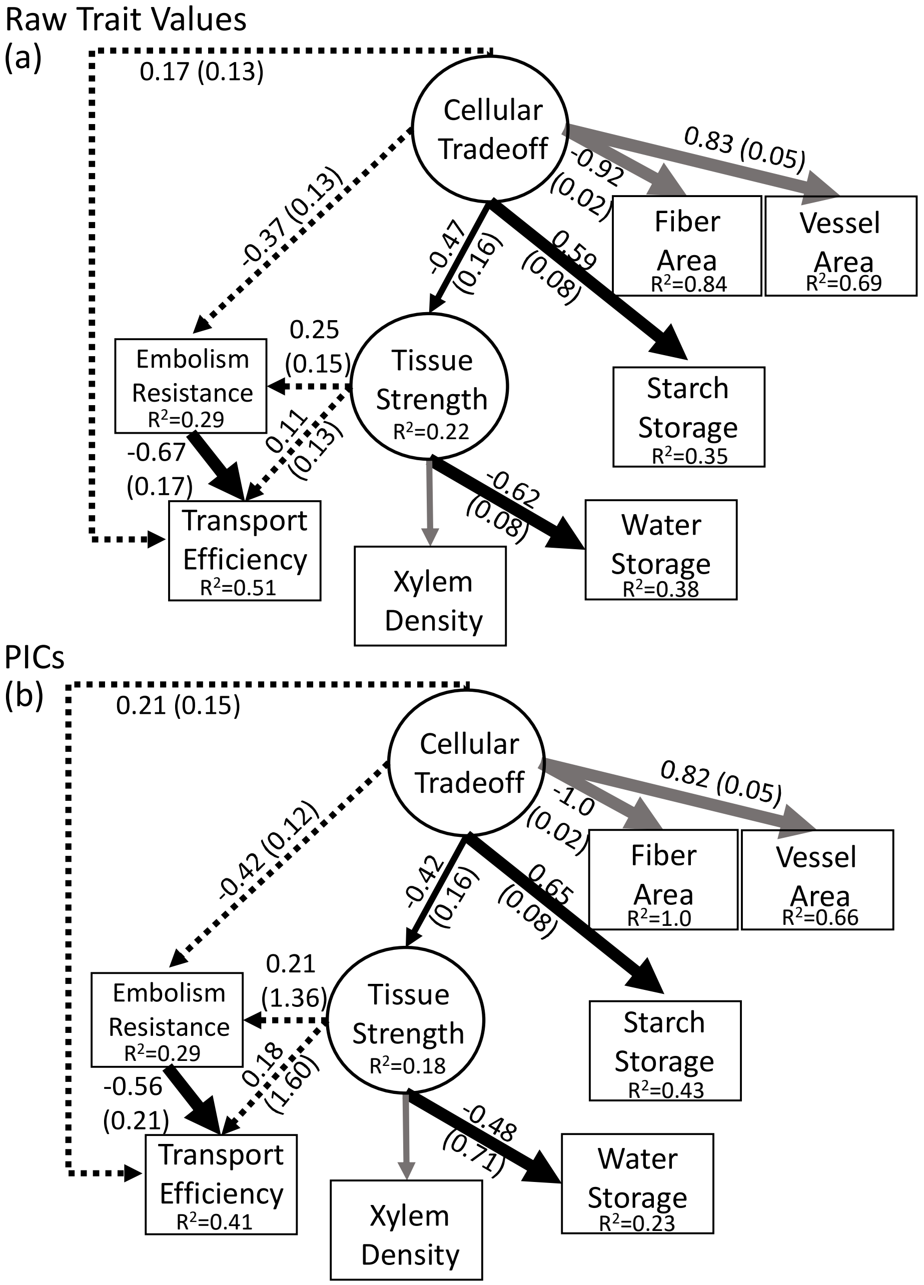

Figure S9. Results from our initial analyzed SEM model for raw trait values (a) and PICs (b) with P_{min} omitted from the model (Table S3 has model fit statistics). The weights of the solid arrows correspond to P-values where the thickest is <0.001, intermediate <0.01, and thinnest is <0.05. Dotted arrows correspond to P>0.05. Values shown along paths are standardized coefficients and standard errors in parentheses (Figure S11 shows unstandardized coefficients). The variance explained (R^2) is shown for each trait. Latent variables are connected to their measured traits by gray arrows. Values are not shown for xylem density because this trait is included within the "tissue strength" variable and the values there apply to xylem density.

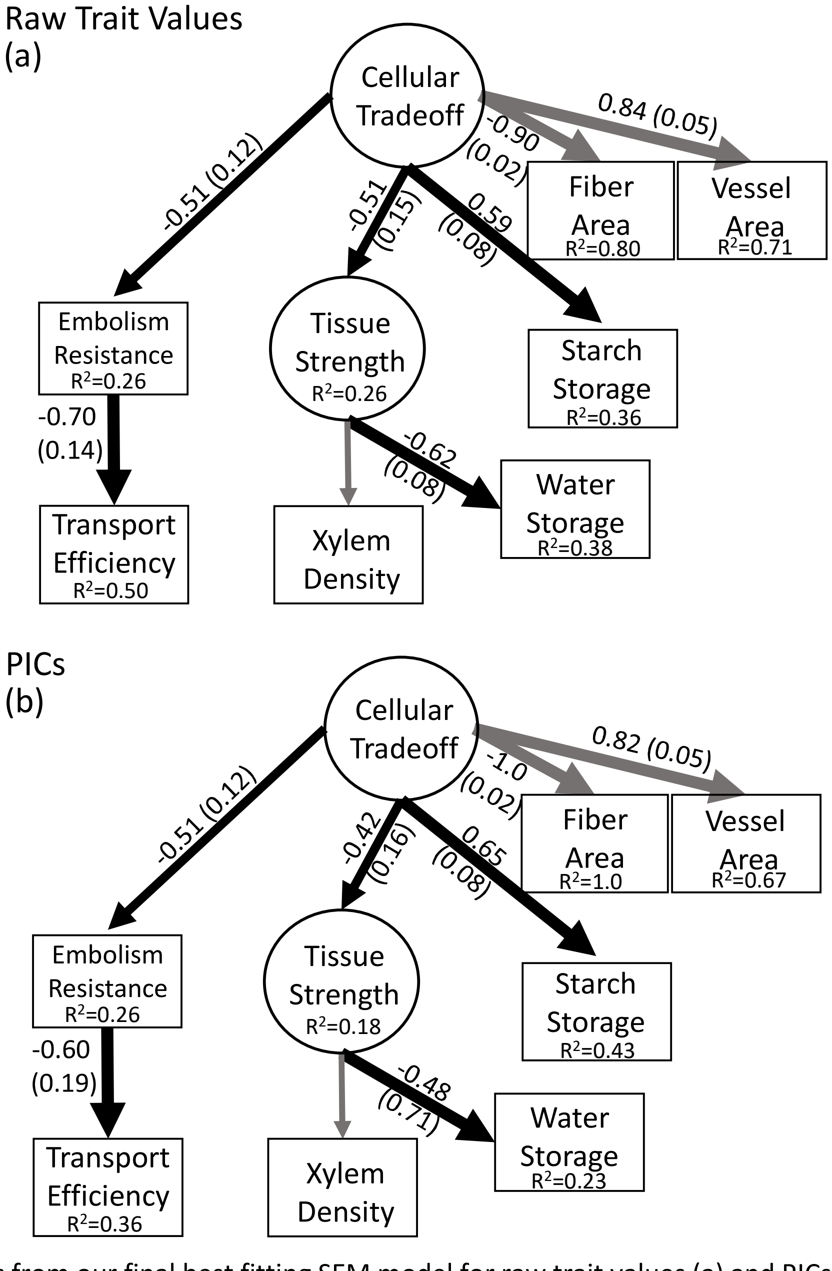

Figure S10. Results from our final best fitting SEM model for raw trait values (a) and PICs (b) with P_{min} omitted from the model (Table S3 has model fit statistics). The weights of the solid arrows correspond to P-values where the thickest is <0.001, intermediate <0.01, and thinnest is <0.05. Dotted arrows correspond to P>0.05. Values shown along paths are standardized coefficients and standard errors in parentheses (Figure S11 shows unstandardized coefficients). The variance explained (R^2) is shown for each trait. Latent variables are connected to their measured traits by gray arrows. Values are not shown for xylem density because this trait is included within the "tissue strength" variable and the values there apply to xylem density.

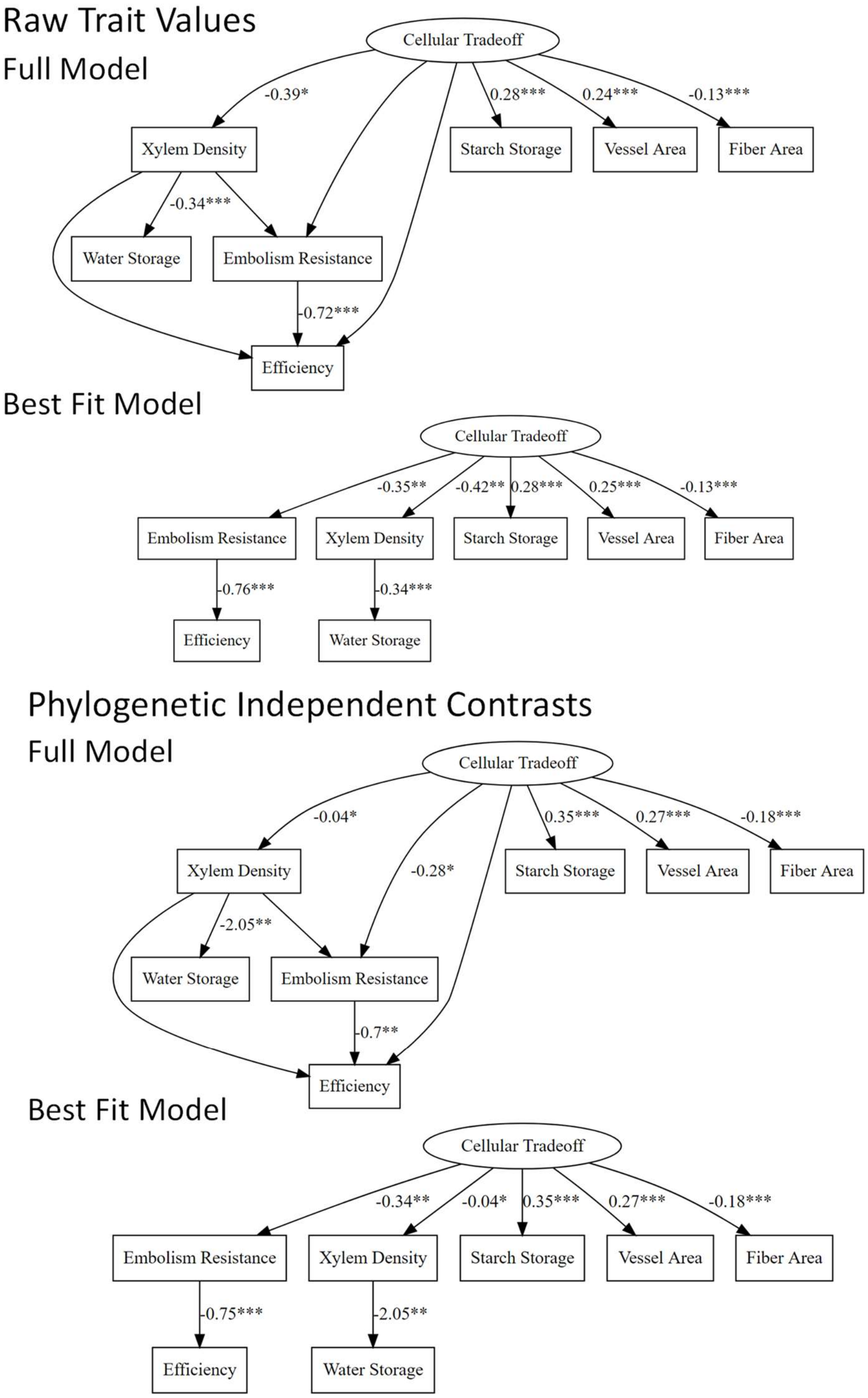

Figure S11. Results from our initial and best fitting SEM models for raw trait values and PICs with P_{min} omitted from the model (Table S3 has model fit statistics). Values shown along paths are unstandardized coefficients and P-values are represented by asterisks (***<0.001; **<0.01, *<0.05). These models do not include "tissue strength" because this function is represented by xylem density within these models.

SI References

1. R. B. Pratt, A. L. Jacobsen, M. I. Percolla, M. E. De Guzman, C. A. Traugh, M. F. Tobin, Starch storage capacity of sapwood is related to dehydration avoidance during drought. *American Journal of Botany* **108**, 91-101 (2021).
2. R. B. Pratt, A. L. Jacobsen, Conflicting demands on angiosperm xylem: Tradeoffs among storage, transport and biomechanics. Plant Cell Environ. **40**, 897–913 (2017).
